# Food Insecurity and Sleep Disturbance Among 223,561 Adolescents: A Multi-Country Analysis of Cross-Sectional Surveys

**DOI:** 10.3389/fpubh.2021.693544

**Published:** 2021-10-01

**Authors:** Qiuying Wang

**Affiliations:** Educational Science Research Institute of Shenzhen, Shenzhen, China

**Keywords:** food insecurity, hunger, sleep, adolescents, global issue

## Abstract

This study was to investigate the association between food insecurity (FI) and sleep disturbance among adolescents. Based on the cross-sectional data of the Global School-based Student Health Survey, this study analyzed self-reported data from adolescents in 68 countries. Multivariate logistic regression and meta-analysis were used to evaluate the association between FI and sleep disturbance. The final sample included 223,561 adolescents. The prevalence of severe FI and sleep disturbance was 6.4% and 8.0%, respectively. Severe FI was significantly associated with a higher risk of sleep disturbance in 48 of the 68 countries after adjusting for covariates, with a pooled OR (95% CI) of 1.94 (1.79–2.09). Overall, the association between FI and sleep disturbance was similar across countries and gender, though a large level of heterogeneity existed across upper- and middle-income countries. Identifying adolescents suffering from FI and remedying the FI severity may be important to improve sleep quality in global adolescents.

## Introduction

Food insecurity (FI), defined as limited or uncertain availability of safe and nutritious foods, or access to food in a socially acceptable manner (e.g., without resorting to the emergency food supply, scavenging, or stealing) (1), can result in individual hunger and subsequent health issues (2). FI is a global problem, and the rate of global FI is currently estimated at 27% (3). Notably, the rate is even higher among adolescents than that of other subpopulations (4–6). According to a study using data from the Global School-based Student Health Survey (GSHS), the overall prevalence of moderate to severe FI among adolescents was 44.9 and 6.2%, respectively (7). An increasing number of studies have revealed the unfavorable health consequences of FI on adolescents' physical and mental health. Adolescents exposed to FI are not only prone to malnutrition due to inadequate nutrient intake (8), but also face greater risks of a myriad of physical (e.g., obesity and asthma) and mental health (e.g., depression and anxiety) problems (9–11). All of these may damage the overall well-being of adolescents and their future development.

There is also a considerable body of research showing that sleep plays an important role in the healthy growth and development of adolescents, including neurocognitive, emotional, behavioral aspects (12–15). Conversely, sleep disturbance is associated with an array of detrimental health outcomes in adolescents, such as poor academic performance, cognitive function, mood regulation (16–19). More seriously, sleep disturbance may also increase the risk of suicidal ideation and suicide attempts in adolescents (20, 21). Nevertheless, previous research has indicated a high prevalence of sleep disturbance among adolescents (22). For example, a study found that the prevalence of sleep disturbance among 84,988 adolescents ranged from 21.1 to 38.8% (23). All of these highlights the severity of sleep disturbance among adolescents, which needs immediate and effective interventions. Before implementing efficient interventions, it is necessary to understand the correlates and determinants of adolescent sleep disturbance.

According to a biopsychosocial and contextual model, adolescents' sleep is affected by biological, psychosocial, and contextual factors (24). Among these factors, low family socioeconomic status and unhealthy dietary patterns are the common correlates explored in adolescent sleep disturbance (23, 25, 26). Of note, FI is often used as an indicator of individuals' low socioeconomic status and insufficient nutrient intake (4, 9, 27). Indeed, using nationally representative cross-sectional data from GSHS, FI was found to be associated with sleep disturbance in Benin and Lebanon adolescents (28, 29). Compared with the evidence from adolescents (30–34), there are relatively more studies on the relationship between FI and sleep disturbance in adults. For example, a study of 14,786 U.S. young adults found that FI was linked to poorer sleep outcomes (e.g., difficulty falling asleep and staying asleep) (34). Another study also suggested that more severe FI was associated with shorter actigraphy-assessed sleep duration and poorer subjective sleep quality (30). Moreover, a systematic review and meta-analysis demonstrated that FI was associated with an increased risk of sleep disorders in adults (35). Based on the evidence from adults-based research, it thus can be expected that FI may be an overlooked risk factor for sleep disturbance in adolescents. The scarcity of research on this topic limits our comprehensive understanding of adolescents' sleep, which urgently indicates that more research is needed to give insights into the association between FI and sleep disturbance among adolescents.

Some research gaps in the literature should be addressed. Most of the existing studies on the relationship between FI and sleep were conducted in a single country. But notably, the impact of FI is worldwide and thus multi-country research is needed. Considering that the association between FI and sleep disturbance may vary between countries owing to the different levels of FI, multinational research is important as they can clarify whether such an FI-sleep association exists universally among countries. Therefore, we need to investigate the association between FI and sleep disturbance from a global perspective. To achieve this purpose, this study used a nationally representative sample of adolescents (the GSHS data) from 68 countries with various income levels, including to investigate (1) the association between FI and sleep disturbance (overall and by gender), and (2) whether the association varies across countries.

## Materials and Methods

### Source of Data

Public data from the GSHS were used. The core objective of the GSHS was to assess and quantify the risk and protective factors of major non-communicable diseases among adolescents. For more information about the GSHS, please visit http://www.who.int/chp/gshs as well as http://www.cdc.gov/gshs. Detailed study design and sampling procedure were also clearly illustrated in the published literature (36–38). Surveys in the GSHS were approved by national government authorities (usually the Ministry of Health or Education) and institutional review committees or ethics committees in each country. Surveys were voluntary and anonymous to protect participants' privacy, with informed consent from students, parents, and/or school officials as appropriate.

Datasets containing variables related to this study were selected from GSHS for analysis. If there were more than two datasets on the same country, chose the latest one. Finally, 68 countries were included in the study. Data for all countries were nationally representative except for several countries where surveys were conducted in selected areas. According to the World Bank classification, 68 countries included were divided into 8 low-income countries, 29 lower- and middle-income countries, 19 upper-and middle-income, and 12 high-income countries. Table 1 lists the characteristics of each country.

**Table 1:** Prevalence of food insecurity and sleep disturbance by country.

**Country**	**Year**	**Response rate (%)**	***N***	**Food insecurity (%)**					**Sleep disturbance (%)**
				**Never**	**Rarely**	**Sometimes**	**Most of the time**	**Always**	
Afghanistan	2014	97	1,858	48.2 (40.3–56.2)	13.0 (8.5–19.4)	18.4 (16.2–20.9)	14.8 (11.2–19.4)	5.5 (3.2–9.2)	22.2 (18.2–26.8)
Antigua and Barbuda	2009	67	1,045	55.7 (52.5–58.9)	14.6 (11.9–17.8)	22.8 (20.2–25.7)	4.6 (3.4–6.3)	2.3 (1.2–4.1)	14.0 (11.4–17.2)
Argentina	2012	71	24,982	64.9 (63.8–65.9)	20.1 (19.3–21.0)	11.5 (10.7–12.5)	2.0 (1.6–2.4)	1.5 (1.2–1.8)	8.6 (7.9–9.4)
Bahamas	2013	78	1,158	52.5 (48.7–56.3)	18.8 (15.3–22.9)	22.0 (19.7–24.6)	5.0 (3.7–6.6)	1.6 (1.0–2.6)	14.1 (11.8–16.8)
Bahrain	2016	89	6,529	44.0 (41.3–46.8)	23.2 (21.5–25.1)	22.1 (20.5–23.7)	8.5 (7.5–9.6)	2.2 (1.7–2.9)	16.1 (13.8–18.7)
Bangladesh	2014	91	2,737	38.0 (33.2–43.1)	8.2 (5.6–11.8)	40.9 (36.2–45.8)	6.1 (4.1–9.0)	6.8 (4.5–10.1)	4.1 (3.2–5.3)
Belize	2011	88	1,411	47.3 (40.1–54.7)	23.3 (19.8–27.2)	15.1 (12.4–18.2)	5.4 (3.8–7.6)	8.9 (6.4–12.3)	18.0 (14.4–22.2)
Bolivia	2012	88	3,071	38.6 (35.6–41.7)	34.7 (32.9–36.6)	18.3 (16.7–20.1)	3.9 (3.1–4.8)	4.5 (3.4–5.8)	6.9 (6.0–7.8)
Brunei Darussalam	2014	65	2,374	38.7 (36.7–40.7)	29.5 (27.8–31.4)	25.5 (23.3–27.7)	5.0 (4.2–5.9)	1.3 (0.9–2.0)	10.2 (8.9–11.7)
Cambodia	2013	85	2,766	46.9 (42.0–51.9)	20.1 (17.6–22.8)	26.7 (23.1–30.6)	3.4 (2.3–5.0)	2.9 (2.1–4.0)	5.0 (4.2–5.9)
Chile	2004	60	1,898	74.8 (72.7–76.8)	17.7 (15.6–20.0)	5.7 (4.7–7.0)	1.2 (0.8–1.8)	0.6 (0.4–0.8)	9.4 (8.3–10.7)
China	2003	98	2,156	59.3 (56.4–62.2)	27.8 (25.7–29.9)	10.8 (9.1–12.8)	1.6 (1.2–2.3)	0.5 (0.2–1.0)	4.6 (3.7–5.6)
Costa Rica	2009	72	2,521	79.8 (76.9–82.4)	12.7 (10.8–14.9)	6.3 (5.2–7.5)	0.6 (0.4–1.0)	0.6 (0.4–1.1)	4.7 (3.8–5.7)
Curacao	2015	83	1,946	76.3 (74.0–78.4)	11.6 (9.9–13.5)	9.0 (7.7–10.6)	2.0 (1.5–2.8)	1.1 (0.7–1.8)	10.7 (9.2–12.4)
Djibouti	2017	83	1,245	54.0 (50.2–57.9)	8.6 (6.8–10.7)	19.5 (17.0–22.3)	5.8 (4.6–7.3)	12.1 (9.7–14.9)	13.9 (11.3–17.0)
Dominica	2016	84	1,175	65.6 (59.6–71.1)	20.6 (17.0–24.6)	12.4 (9.5–15.9)	0.7 (0.4–1.3)	0.7 (0.4–1.5)	9.8 (7.8–12.3)
Ecuador	2007	84	1,930	60.2 (57.5–52.8)	28.7 (26.2–31.3)	6.9 (5.9–8.0)	1.9 (1.3–2.7)	2.4 (1.6–3.5)	8.9 (7.3–10.8)
Egypt	2006	85	3,443	59.6 (52.8–66.1)	22.2 (17.6–27.6)	13.6 (10.1–18.1)	3.0 (2.1–4.3)	1.5 (0.9–2.4)	8.9 (7.3–10.8)
El Salvador	2013	88	1,740	65.8 (62.3–69.2)	19.8 (17.4–22.5)	11.2 (9.4–13.3)	1.3 (0.9–1.9)	1.8 (1.3–2.5)	6.9 (5.7–8.3)
Fiji	2016	79	2,662	42.0 (37.9–46.1)	9.7 (8.1–11.6)	38.3 (34.5–42.1)	7.9 (6.4–9.7)	2.2 (1.7–2.9)	11.4 (10.2–12.3)
French Polynesia	2015	70	2,625	36.2 (33.1–39.5)	32.3 (30.3–34.4)	21.3 (19.5–23.2)	8.0 (6.8–9.2)	2.2 (1.6–3.1)	11.2 (10.2–12.3)
Ghana	2012	71	1,262	35.5 (29.4–42.1)	5.7 (4.1–7.9)	44.6 (38.7–50.6)	7.9 (6.5–9.7)	6.3 (4.0–9.7)	12.9 (10.5–15.7)
Grenada	2008	78	1,220	57.9 (54.5–61.3)	14.5 (11.8–17.7)	20.7 (17.9–23.8)	5.1 (3.6–7.2)	1.8 (1.2–2.8)	11.0 (8.6–14.0)
Guatemala	2015	82	3,513	63.0 (58.2–67.6)	23.0 (20.0–26.4)	11.3 (8.4–15.1)	1.2 (0.7–2.1)	1.5 (0.8–2.7)	5.8 (4.6–7.4)
Guyana	2010	76	2,086	55.2 (50.9–59.4)	12.4 (9.4–16.1)	25.2 (20.9–30.1)	4.0 (3.2–5.1)	3.2 (1.6–6.2)	13.8 (12.2–15.7)
Honduras	2012	79	1,576	64.2 (60.7–67.5)	22.1 (19.8–24.6)	10.1 (8.8–11.6)	1.6 (0.9–2.7)	2.0 (1.6–2.6)	5.0 (4.1–6.1)
Indonesia	2015	94	9,615	44.9 (42.5–47.4)	12.4 (11.2–13.8)	38.8 (36.9–40.7)	2.7 (2.3–3.1)	1.2 (0.9–1.6)	4.4 (3.8–5.1)
Iraq	2012	88	1,720	67.0 (62.4–71.3)	17.1 (14.5–20.1)	7.6 (6.1–9.4)	2.8 (2.1–3.9)	5.4 (3.8–7.7)	12.2 (9.7–15.3)
Jamaica	2017	60	1,410	52.4 (48.2–56.6)	20.7 (17.4–24.4)	20.8 (17.8–24.2)	4.4 (3.4–5.7)	1.6 (1.0–2.6)	13.0 (11.1–15.0)
Jordan	2007	100	1,742	51.9 (47.7–56.1)	21.4 (19.2–23.8)	14.3 (12.3–16.5)	7.8 (6.2–9.7)	4.7 (3.6–6.0)	18.0 (15.2–21.2)
Kenya	2004	84	2,417	39.2 (33.4–45.4)	14.8 (12.2–17.8)	31.9 (27.6–36.6)	10.5 (8.2–13.3)	3.6 (2.5–5.0)	15.1 (12.6–18.0)
Kuwait	2015	78	2,713	52.1 (49.2–55.0)	22.6 (20.9–24.3)	18.4 (16.5–20.4)	5.0 (3.9–6.4)	1.9 (1.4–2.6)	19.5 (16.4–23.0)
Lao	2015	70	3,323	51.1 (45.2–57.0)	15.7 (13.9–17.7)	32.0 (27.6–36.7)	0.9 (0.4–1.7)	0.3 (0.1–1.2)	4.3 (3.4–5.3)
Lebanon	2017	82	4,566	70.8 (68.3–73.1)	18.2 (16.8–19.6)	8.3 (7.1–9.6)	1.9 (1.5–2.3)	0.9 (0.6–1.4)	13.2 (11.8–14.8)
Malaysia	2012	89	23,425	40.9 (39.6–42.2)	28.2 (27.0–29.4)	26.3 (25.3–27.3)	2.5 (2.2–2.8)	2.1 (1.8–2.5)	5.1 (4.7–5.5)
Mauritania	2010	70	1,693	41.5 (37.4–45.8)	31.4 (28.7–34.2)	17.4 (15.0–20.0)	5.4 (4.4–6.6)	4.4 (2.5–7.6)	11.2 (8.7–14.3)
Mauritius	2017	84	2,634	57.6 (54.4–60.7)	19.1 (17.4–20.9)	16.6 (14.6–18.7)	4.3 (3.1–6.0)	2.5 (1.5–3.9)	8.6 (7.4–10.0)
Mongolia	2013	88	4,830	63.4 (61.2–65.5)	22.9 (21.2–24.7)	12.1 (10.7–13.7)	0.8 (0.6–1.2)	0.8 (0.5–1.1)	5.2 (4.5–6.1)
Morocco	2016	91	5,020	67.7 (65.4–69.9)	11.9 (9.8–14.3)	12.0 (10.8–13.5)	5.9 (5.1–6.7)	2.5 (1.9–3.4)	15.8 (14.3–17.3)
Mozambique	2015	80	1,092	54.6 (44.1–64.7)	9.3 (5.9–14.5)	25.8 (19.4–33.4)	4.1 (2.3–7.5)	6.1 (4.3–8.6)	8.5 (6.3–11.4)
Myanmar	2016	86	2,487	69.5 (66.7–72.1)	6.4 (4.8–8.5)	21.8 (20.0–23.8)	1.2 (0.8–1.8)	1.1 (0.6–2.0)	3.3 (2.7–4.1)
Namibia	2013	89	2,883	48.1 (43.6–52.5)	5.5 (1.2–7.1)	37.0 (32.6–41.7)	6.7 (5.5–8.1)	2.7 (2.1–3.6)	13.6 (11.8–15.7)
Nepal	2015	69	5,666	67.4 (63.0–71.6)	5.2 (4.4–6.2)	23.5 (20.0–27.3)	2.1 (1.3–3.2)	1.8 (0.9–3.7)	4.1 (3.3–5.1)
Oman	2015	92	2,914	63.7 (61.1–66.3)	17.7 (16.1–19.6)	14.4 (12.7–16.2)	2.7 (2.2–3.4)	1.4 (1.0–2.1)	17.9 (15.9–20.1)
Pakistan	2009	76	4,490	75.5 (70.0–80.3)	6.4 (4.9–8.1)	13.1 (10.0–16.9)	2.2 (1.6–3.0)	2.9 (2.2–3.7)	7.6 (6.5–9.0)
Paraguay	2017	87	2,663	71.9 (69.3–74.3)	17.5 (15.2–20.0)	8.7 (7.4–10.1)	0.8 (0.6–1.1)	1.2 (0.8–1.9)	8.7 (7.2–10.5)
Philippines	2015	79	7,794	30.3 (28.1–32.5)	32.3 (30.2–34.5)	30.2 (28.1–32.4)	4.5 (3.8–5.3)	2.7 (2.2–3.4)	10.6 (9.7–11.7)
Qatar	2011	87	1,381	70.2 (66.3–73.8)	14.9 (12.8–17.2)	8.2 (6.6–10.0)	4.2 (3.2–5.4)	2.6 (1.7–3.9)	17.5 (15.0–20.4)
Saint Vincent and the Grenadines	2007	84	1,056	60.3 (56.7–63.8)	11.1 (8.6–14.2)	22.0 (18.4–26)	3.7 (2.6–5.2)	2.9 (1.9–4.3)	13.2 (11.2–15.5)
Samoa	2017	59	1,454	25.7 (22.6–29.1)	35.5 (32.8–38.4)	26.1 (22.6–29.8)	4.9 (3.7–6.4)	7.9 (5.7–10.7)	9.1 (7.6–10.9)
Seychelles	2015	82	2,124	56.7 (53.6–59.7)	15.7 (13.7–17.9)	15.6 (13.9–17.5)	7.6 (5.9–9.7)	4.4 (3.5–5.6)	11.3 (9.9–12.8)
Solomon Islands	2011	85	1,094	15.1 (12.1–18.6)	8.6 (5.7–12.8)	66.0 (59.8–71.7)	7.8 (5.6–10.8)	2.5 (1.4–4.5)	13.0 (10.9–15.5)
Sri Lanka	2016	89	3,075	72.0 (68.2–75.5)	13.0 (11.0–15.3)	12.1 (8.5–16.8)	1.0 (0.7–1.5)	1.9 (1.4–2.6)	4.6 (3.5–5.8)
St. Lucia	2007	82	1,180	56.0 (52.9–59.0)	17.6 (15.0–20.4)	20.1 (17.3–23.3)	4.9 (3.8–6.2)	1.4 (0.9–2.3)	11.1 (9.4–13.0)
Suriname	2016	83	1,759	57.6 (52.9–62.1)	11.4 (9.6–13.4)	20.4 (16.8–24.5)	5.4 (4.6–6.3)	5.3 (4.3–6.6)	12.1 (10.4–14.1)
Tanzania	2006	87	1,667	73.9 (69.1–78.2)	15.7 (12.3–19.8)	6.9 (5.6–8.5)	2.2 (1.4–3.5)	1.3 (0.9–2.0)	3.2 (2.4–4.2)
Thailand	2015	89	4,817	47.5 (44.6–50.5)	24.0 (22.3–25.7)	24.9 (22.5–27.4)	1.9 (1.5–2.4)	1.7 (1.2–2.4)	8.1 (6.7–9.8)
Timor–Leste	2015	79	2,454	50.4 (47.9–52.8)	13.6 (11.9–15.5)	25.3 (22.6–28.1)	4.6 (3.8–5.4)	6.2 (4.9–7.8)	11.1 (9.4–13.0)
Tonga	2017	90	2,608	35.0 (32.3–37.7)	25.8 (24.0–27.5)	28.9 (27.0–31.0)	3.9 (3.1–4.8)	6.5 (5.4–7.8)	14.0 (12.4–15.7)
Trinidad and Tobago	2017	89	3,233	49.1 (46.3–52.0)	22.7 (20.3–25.3)	20.3 (18.6–22.1)	5.3 (4.4–6.4)	2.6 (2.0–3.3)	13.7 (12.2–15.4)
Tunisia	2008	83	2,301	59.1 (55.3–62.9)	15.2 (13.4–17.2)	18.1 (15.8–20.5)	5.5 (4.3–7.0)	2.2 (1.6–3.0)	18.6 (16.3–21.0)
Uganda	2003	69	2,560	46.7 (41.3–52.2)	11.9 (9.9–14.2)	33.1 (28.6–38.0)	4.2 (3.2–5.6)	4.1 (2.6–6.4)	10.3 (8.8–12.0)
United Arab Emirates	2016	80	4,907	54.1 (50.9–57.4)	19.9 (18.2–21.6)	18.0 (16.4–19.7)	6.0 (5.0–7.1)	2.1 (1.6–2.6)	15.6 (14.1–17.2)
Uruguay	2012	77	3,156	76.8 (74.6–78.9)	16.2 (14.5–18.0)	5.7 (4.7–6.9)	0.8 (0.5–1.3)	0.6 (0.4–0.9)	5.8 (5.0–6.9)
Vanuatu	2016	57	1,765	38.9 (35.4–42.5)	4.5 (2.6–7.6)	48.4 (44.8–52.0)	5.3 (4.0–7.0)	2.9 (1.4–3.4)	6.5 (5.3–8.1)
Venezuela	2003	85	1,820	78.7 (74.2–82.5)	9.3 (8.0–10.7)	9.0 (6.7–12.1)	0.8 (0.4–1.8)	2.2 (1.4–3.4)	3.3 (2.6–4.1)
Yemen	2014	75	1,918	43.4 (37.3–49.8)	24.6 (21.3–28.3)	21.8 (17.8–26.3)	7.6 (6.2–9.2)	2.6 (1.7–3.9)	14.1 (11.8–16.8)
Zambia	2004	70	1,206	18.0 (14.7–22.0)	8.3 (6.2–11.0)	45.6 (42.6–48.6)	19.7 (16.8–22.9)	8.4 (6.4–10.9)	24.4 (21.3–27.7)
Total			223,561	50.0	17.1	26.5	3.7	2.7	8.0

### Study Variables

#### Food Insecurity

FI was assessed by the question “During the past 30 days, how often did you go hungry because there was not enough food in your home?” Answer options were: never, rarely, sometimes, most of the time, and always. For further analyses, a dichotomized variable on FI was used. Participants who answered “most of the time” and “always” were considered to have severe FI (reduced food intake and disrupted eating patterns) (39).

#### Sleep Disturbance

The assessment question for sleep disturbance was “During the past 12 months, how often have you been so worried about something that you could not sleep at night?” Answer options were: never, sometimes, most of the time, and always. Consistent with previous research (38, 40, 41), participants who answered “most of the time” or “always” were considered to have sleep disturbance in this study.

#### Covariates

Covariates were selected based on previous studies (36–38) and availability from the datasets, including age, gender, physical activity, sedentary behavior, and bullying victimization. Physical activity was evaluated with the item: “In the past 7 days, how many days were you physically active for at least 60 min per day?” The answers ranged from 0 to 7 days. Sedentary behavior was assessed with the item: “How much time do you spend sitting and watching television, playing computer games, chatting with friends, or doing other sedentary activities (not including time at school or doing homework) on a typical or normal day?” The answer options were: <1, 1–2, 3–4, 5–6, 7–8, or ≥8 h per day. Bullying victimization was defined as being bullied on at least 1 day in the last 30 days.

### Statistical Analyses

The overall and country-specific prevalence of FI and sleep disturbance was calculated by descriptive analysis. Multivariate logistic regression analyses were used to analyze the association between FI and sleep disturbance (overall and by gender) after adjusting for gender, age, physical activity, sedentary behavior, bullying victimization, and country (gender-stratified and country-wise analyses were not adjusted for gender and country, respectively). Higgin's *I*^2^ statistics were calculated to evaluate the level of heterogeneity between countries. Generally, values of <40% are considered to be negligible heterogeneity, and 40–60% are moderate (42). By combining the estimates for each country into the random-effects meta-analysis, a pooled estimate was obtained. For the cases of missing data, a complete case analysis was carried out. Taylor linearization method was used to account for the sample weight and clustered study design. Logistic regression analyses results were presented as odds ratios (ORs) with 95% confidence intervals (CIs). The statistical significance level was set at *p* < 0.05. All statistical analyses were performed using Stata 16.1 (Corp Limited).

## Results

The final sample included 223,561 adolescents aged 12–17 years (47.3% boys). The proportions of students in each age group were as follows: 6.7% (12 years); 19.5% (13 years); 24.3% (14 years); 23.1% (15 years); 17.7% (16 years); and 8.6% (17 years). Overall, the prevalence of FI with levels of being rarely, sometimes, most of the time, and always was 17.1, 26.5, 3.7, and 2.7%, respectively. Moreover, 8.0% of the students ever had sleep disturbance in the past 12 months. The prevalence of sleep disturbance and FI varied widely across countries. Specifically, the prevalence of sleep disturbance ranged from 3.2% (Tanzania) to 24.4% (Zambia). Regarding the severity of FI, 0.6% (Costa Rica) to 19.7% (Zambia) participants reported “most of the time,” and 0.3% (Lao) to 12.1% (Djibouti) participants reported “always.” More information about the sample characteristics by each country is provided in Table 1.

**Figure 1:**
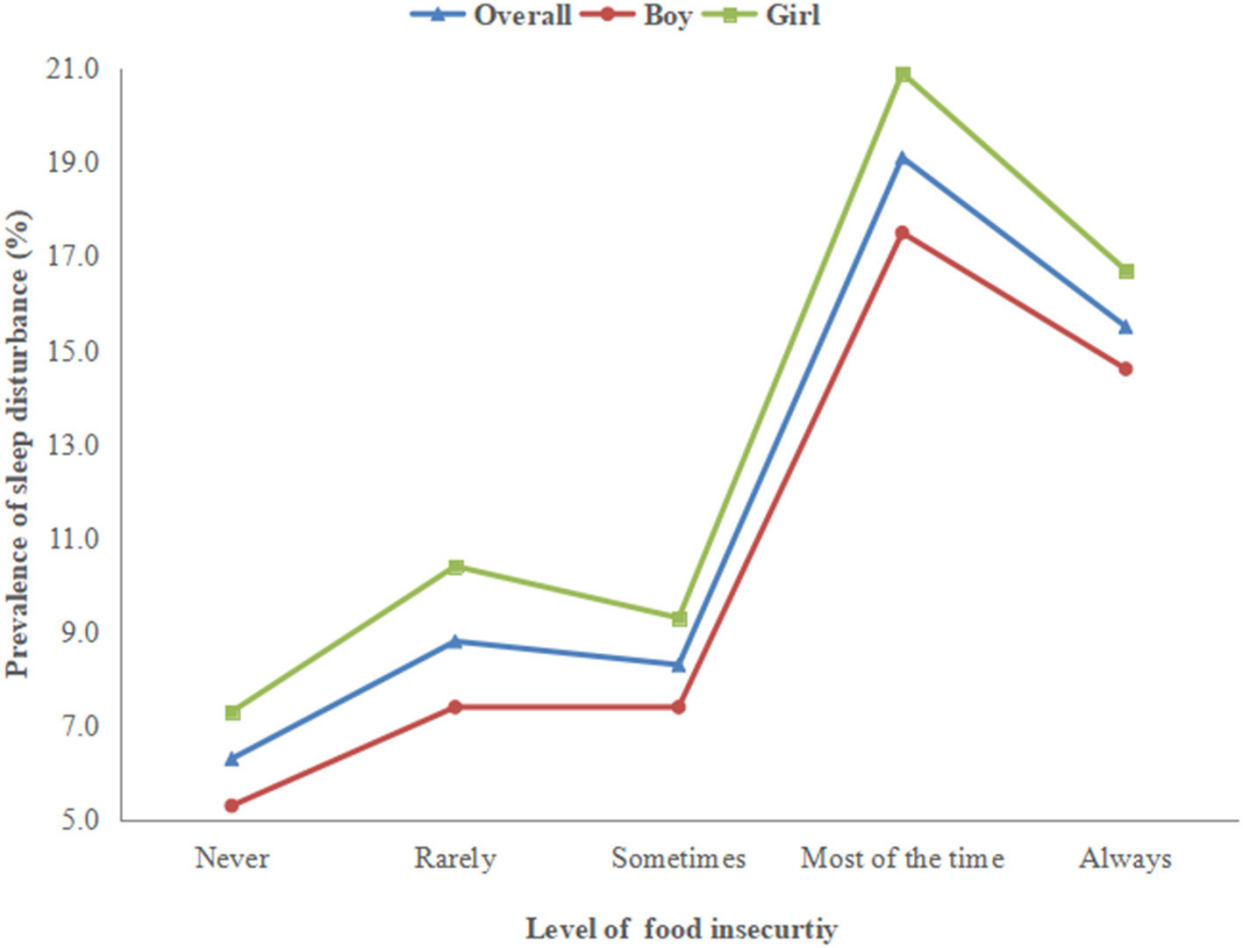
Prevalence of sleep disturbance by levels of food insecurity.

Figure 1 shows the prevalence of sleep disturbance corresponding to the severity of FI. Specifically, compared to those with less severe FI (never/rarely/sometimes), the prevalence of sleep disturbance was much higher among adolescents with severe FI in the overall and gender-stratified sample.

Table 2 details the association between FI and sleep disturbance in the fully adjusted model. Adolescents with any levels of FI (rarely/sometimes/most of the time/always hungry) were more likely to report sleep disturbance than those who had never experienced FI. In particular, being most of the time and always hungry (severe FI) was associated with 2.54 (95% CI = 2.23–2.90) and 2.29 (95% CI = 1.93–2.71) times higher odds for sleep disturbance, respectively. A similar significant relationship was observed in both boys and girls. More details can be found in Table 2.

**Table 2:** Association between food insecurity and sleep disturbance estimated by multivariable logistic regression (overall and by gender).

**Level of food insecurity**	**OR (95% CI)**		
	**Overall** ^**a**^	**Boy** ^**b**^	**Girl** ^**b**^
Rarely	1.23 (1.13–1.35)	1.24 (1.07–1.43)	1.25 (1.11–1.40)
Sometimes	1.38 (1.26–1.51)	1.39 (1.19–1.61)	1.38 (1.22–1.55)
Most of the time	2.54 (2.23–2.90)	2.82 (2.33–3.41)	2.32 (1.94–2.78)
Always	2.29 (1.93–2.71)	2.43 (1.86–3.16)	2.21 (1.74–2.81)

*OR, Odds ratio; CI, Confidence interval*.

a *Adjusted for age, gender, physical activity, sedentary behavior, and bullying victimization, and country*.

b *Adjusted for age, physical activity, sedentary behavior, and bullying victimization, and country*.

**Figure 2:**
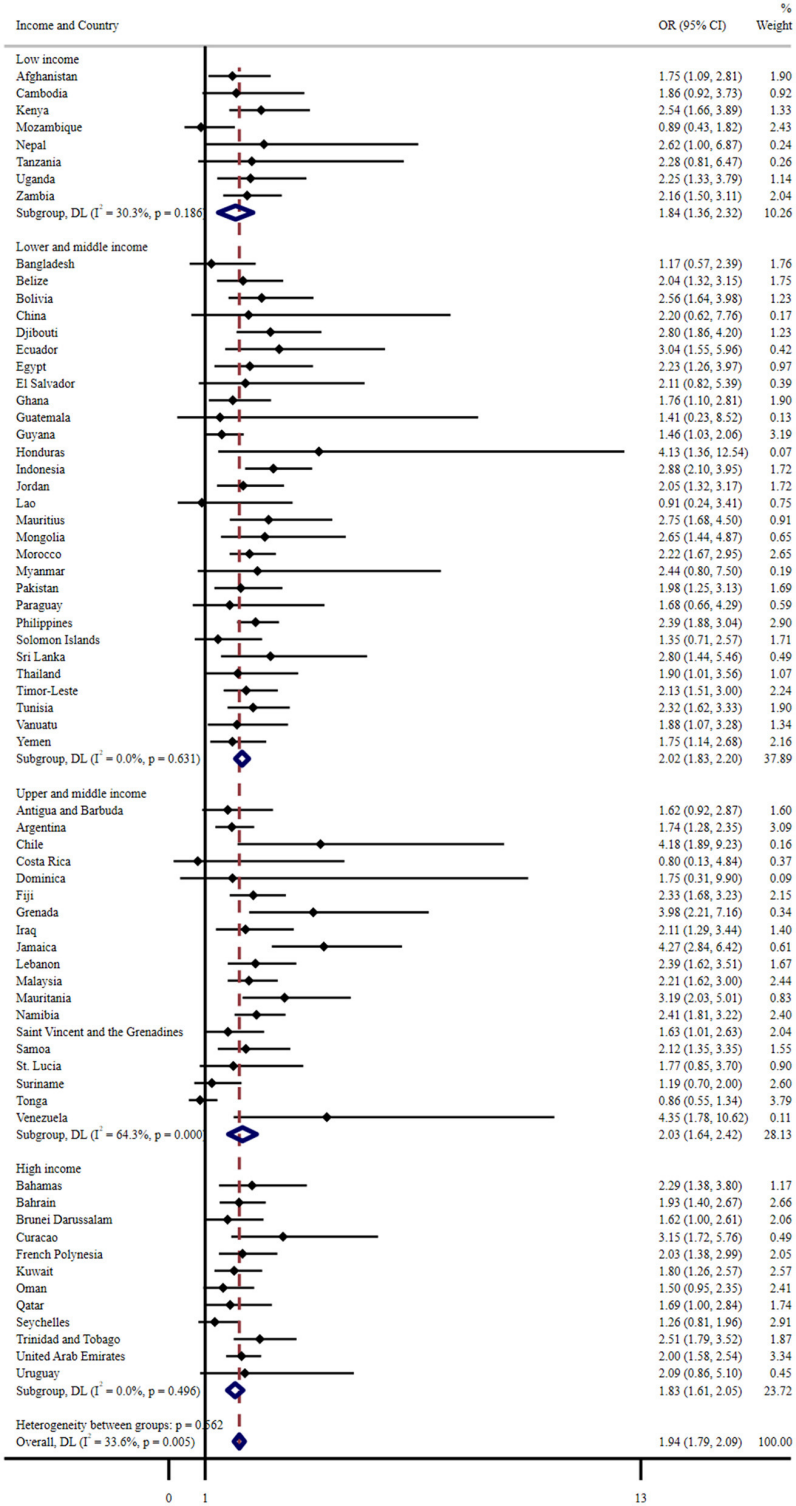
Country-wise association between severe food insecurity (most of the time/always hungry) and sleep disturbance estimated by multivariable logistic regression. OR, Odds ratio; CI, Confidence interval. Reference category is no food insecurity (never, rarely, sometimes). Models are adjusted for age, gender, physical activity, sedentary behavior, and bullying victimization. Overall estimates were obtained by meta-analysis with random effects.

Figure 2 shows the results of the country-wise multivariate logistic regression analysis. Compared to less severe FI (never/rarely/sometimes), severe FI (most the time or always hungry) was significantly associated with a higher risk of sleep disturbance in 48 of the 68 countries. Overall, the pooled OR was 1.94 (1.79–2.09), and the heterogeneity between countries was negligible (*I*^2^ = 33.6%). In upper- and middle-income countries, a large level of heterogeneity (*I*^2^ = 64.3%) was observed in the association between FI and sleep disturbance.

## Discussion

### Main Findings

Based on a large global adolescent sample, this study complements the emerging literature on FI as a potential social determinant of sleep disturbance. Overall, 6.4% of adolescents experienced severe FI. Sleep disturbance was also common, affecting 8.0% of adolescents in this representative sample. The prevalence of sleep disturbance increased with the severity of FI. After adjusting for covariates, severe FI was significantly associated with a higher risk of sleep disturbance in 48 of the 68 countries, with a pooled OR being 1.94 (1.79–2.09). In general, the association was similar across countries and genders, though there was a great degree of heterogeneity in upper- and middle-income countries.

### Interpretation of Findings

Although the prevalence of FI and sleep disturbance is difficult to compare with other studies due to different definitions and measures adopted across studies, the prevalence of FI and sleep disturbance in this study is within the estimated range of previous reports (43, 44). More importantly, the results of this study support prior evidence showing associations between FI and sleep disturbance (e.g., short sleep duration and poor sleep quality) (30, 34). Based on the aforementioned biopsychosocial and contextual model of adolescent sleep (24), several theoretically plausible mechanisms may link FI to sleep disturbance, acting via multiple, interlinked biological, psychosocial, and contextual pathways. That is, FI may affect adolescent sleep by altering sleep-related biological, psychosocial, or contextual factors. The detailed interpretations are as follows.

Regarding biological pathways, for instance, levels of metabolic activity may affect the state of vigilance, promoting wakefulness and hunger at low levels, or sleep and satiety at high levels (45). Therefore, hunger, a direct outcome of FI, often coexists with wakefulness, leading to problems in sleep. FI is also a significant source of chronic stress, may influence stress-related physiological arousal dysfunction and raise cortisol levels, which are implicated in sleep disturbance (34). Besides, adolescents with FI tend to suffer from nutritional deficiencies and obesity, which can affect slow-wave sleep and rapid eye movement (46). In terms of psychosocial pathways, adolescents with awareness of FI in their family may have feelings of alienation and relative deprivation, leading to low self-efficacy and helplessness, which is negatively related to psychological functioning (47). Subsequently, the resulting mental distress can adversely affect the sleep of adolescents as many studies have found the bidirectional relationship between mental disorders and sleep disturbance (48, 49). Indeed, evidence from adults has shown that mental health problems could mediate the association between FI and poor sleep quality (32). As for the contextual factors, the reason behind FI is often poverty, while the dissatisfaction of resource demand caused by poverty typically does not occur in isolation; with a tight household budget, the family may not be able to pay for food, housing, clothing, health care, and other living expenses at the same time (50). Consequently, FI is always related to housing insecurity, and poor housing conditions may link to poor sleep owing to concerns about personal safety, exposure to higher noise levels, and insufficient heating or cooling (33).

The overall heterogeneity of the association between FI and sleep disturbance across all the included countries was negligible, reflecting that this association is common among adolescents around the world. Large inconsistency was noted across the upper- and middle-income countries, indicating that there may be a significant difference in the association between FI and sleep disturbance in the samples from upper- and middle-income countries. Such discrepancy may be explained by the differences in social, cultural, and environmental attributes. Alternatively, heterogeneity between countries may be related to the type of nutritional deficiency caused by severe FI, depending on the settings of countries. A cross-countries comparison on the differences in the association between FI and sleep disturbance is beyond the scope of this study, and future work is needed to clarify these differences.

### Implications for Practice

Although a causal relationship could not be determined in this study, the coexistence of FI and sleep disturbance deserves more attention, as both are related to adverse health outcomes (e.g., depression and suicide ideation). Researchers have utilized the risks and resources model to examine a set of important correlates of FI among adolescents (51), including a series of perceived levels (e.g., individuals, families, schools, peers, and communities). The model may help to understand how to best solve possible problems of the coexistence of FI and sleep disturbance in adolescents and to take targeted intervention strategies.

Moreover, given the bidirectional relationship between sleep disturbance and mental health problems (52, 53), this study suggests that FI may be a potential risk factor for poorer sleep and mental health and calls for more research on sleep and mental health in the context of poverty. FI is an important source of daily stress. Therefore, risk assessments can regularly include the inquiry about dietary patterns, and interventions should be multifaceted to address the need of this aspect. Furthermore, to solve the adverse effects of FI on adolescents, several potentially useful interventions have been proposed, such as family-based programs that provide subsidized nutritional food to food-insecure families and school-based programs that can provide school meals for target students (50, 54). However, more long-term and effective intervention programs still need to be developed and implemented.

### Limitations and Strengths

The present study has several limitations. First, the data used in this study were cross-sectional, so while sleep disturbance was unlikely to drive FI in adolescents, the direction of the association between the two variables was uncertain. Therefore, longitudinal research on this topic is warranted. Second, this study only involves adolescents in schools, so the results cannot infer all adolescents. Third, GSHS relied on self-report, which could have introduced a certain degree of recall bias and desirability bias. Fourth, the measurement of FI was based on a single question, asking about the frequency of hunger caused by lack of food at home. The severity of FI was characterized by the frequency of hunger, but hunger is only a manifestation of FI. Considering this, the classification of FI used in this study may not precisely reflect the true levels of FI. Fifth, in the meta-analysis to explore the association between severe FI (most of the time/always hungry) and sleep disturbance, FI (a multiclass ordinal variable) was dichotomized for analysis. This dichotomy may lead to the loss of some more detailed information. Sixth, sleep disturbance in this study mainly referred to worry-induced sleep disturbance, thus caution should be taken when extrapolating to other sleep disturbances. Finally, data covered in this study were collected in different countries and years, which may have an impact on the results although the country has been listed as a covariate. Despite these limitations, an obvious advantage of this study is that it used a large sample of adolescents from 68 countries to investigate the novel association between FI and sleep disturbance. This study complements the literature in this field and contributes to a broader understanding of the social determinants of sleep.

## Conclusions

FI was prevalent among adolescents in most countries and associated with a higher incidence of sleep disturbance. The coexistence of FI and sleep disturbance in adolescents may be an unrecognized public health problem as both FI and sleep disturbance are associated with adverse health outcomes. Findings point to the importance of solving FI and social inequality to improve the physical and mental health of global youth.

## Data Availability Statement

The raw data supporting the conclusions of this article will be made available by the authors, without undue reservation.

## Ethics Statement

Ethical review and approval was not required for the study on human participants in accordance with the local legislation and institutional requirements. Written informed consent to participate in this study was provided by the participants' legal guardian/next of kin.

## Author Contributions

QW conducted the statistical analyses and wrote the manuscript.

## Conflict of Interest

The author declares that the research was conducted in the absence of any commercial or financial relationships that could be construed as a potential conflict of interest.

## Publisher's Note

All claims expressed in this article are solely those of the authors and do not necessarily represent those of their affiliated organizations, or those of the publisher, the editors and the reviewers. Any product that may be evaluated in this article, or claim that may be made by its manufacturer, is not guaranteed or endorsed by the publisher.
